# A + AVD for Treatment of Hodgkin Lymphoma Variant of Richter's Transformation

**DOI:** 10.1155/2024/7612622

**Published:** 2024-02-24

**Authors:** Benjamin Heyman, Michael Choi, Thomas J. Kipps

**Affiliations:** ^1^Division of Regenerative Medicine, Department of Medicine, UC San Diego Moores Cancer Center, UC San Diego, La Jolla, California 92093, USA; ^2^Division of Hematology/Oncology, Department of Medicine, UC San Diego, La Jolla, California 92093, USA; ^3^Center for Novel Therapeutics, Division of Hematology/Oncology, Department of Medicine, UC San Diego, La Jolla, California 92093, USA

## Abstract

Hodgkin lymphoma variant of Richter's transformation (HvRT) is a rare complication for patients with chronic lymphocytic leukemia (CLL), with an overall poor prognosis. We present the first known case series of patients with HvRT treated with the combination of brentuximab vedotin, doxorubicin, vinblastine, and dacarbazine (A + AVD). In our series of 4 patients, two patients treated with A + AVD for HvRT had durable remissions of 40 and 42 months, while two patients had disease progression and ultimately died. Continued investigation into the optimal management for patients with HvRT is still needed.

## 1. Introduction

Chronic lymphocytic leukemia (CLL) is the most common indolent B-cell lymphoid malignancy [1]. The disease's course can be highly variable with many patients with symptomatic disease at diagnosis requiring initiation of treatment, while others may never require treatment [2]. Treatment of CLL has changed dramatically from the use of chemoimmunotherapy, now to the administration of targeted agents such as Bruton tyrosine kinase inhibitors (BTKi) and B-cell lymphoma 2 inhibitors (BCL-2i) that can produce durable remissions with significantly less toxicity [3–5]. However, despite our advances in the treatment of patients with CLL, a much feared potential complication is Richter's transformation (RT), where CLL transforming into an aggressive lymphoma occurs in approximately 2–10% of patients over the course of their disease [6]. Transformation occurs due to the acquisition of genetic defects that facilitate rapid proliferation and cell growth [7]. In more than 90% of patients, transformation will occur as the diffuse large B-cell lymphoma (DLBCL) variant [8]. However, a minority will transform to Hodgkin lymphoma, termed Hodgkin lymphoma variant of Richter's transformation (HvRT) [9]. In HvRT, the malignant cells have morphological and immunophenotypic features of classic Hodgkin lymphoma (cHL) with Reed–Sternberg (RS) cells [6]. Two types of HvRT have been described. Type 1 is characterized by RS cells scattered in a background of CLL. In type 2 transformation, RS cells are present independent of CLL cells [10]. It is unclear if type 1 and type 2 are truly distinct entities, or rather represent a spectrum of the disease, with Type I pattern being a relatively early event in the evolution of the disease [10, 11]. HvRT is also frequently EBV-positive, which may contribute to its development, especially in patients who have received prior fludarabine-based treatment which can cause prolonged lymphodepletion [10, 12].

Brentuximab vedotin (BV) is a CD30 antibody conjugated by a protease-cleavable linker to the microtubule-disrupting drug monomethyl auristatin E [13]. It has been approved for several indications including both treatment naive and relapsed/refractory (R/R) cHL [13, 14]. The echelon-1 clinical trial established brentuximab vedotin, doxorubicin, vinblastine, and dacarbazine (A + AVD) as a standard of care front-line regimen for patients with advanced stage treatment naive cHL after it was found to have improved 6-year progression-free survival (PFS) and overall survival (OS) when compared to doxorubicin, bleomycin, vinblastine, and dacarbazine (ABVD) [14]. The use of A + AVD has not been previously reported in patients with HvRT. Here, we report a case series of four patients with newly diagnosed HvRT treated with A + AVD.

## 2. Case Series

**Figure 1:**
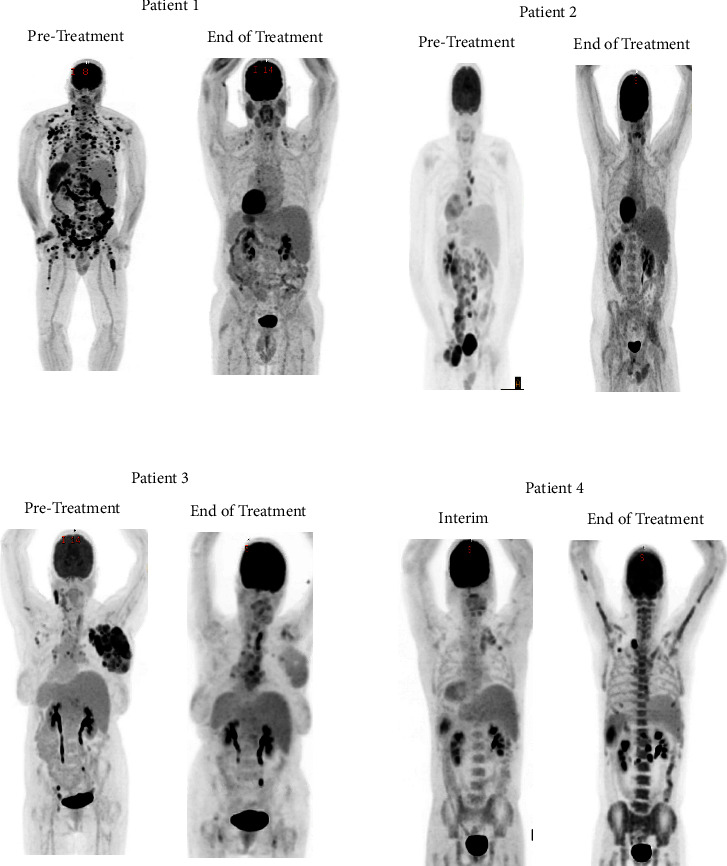
PET/CT response assessments.

Here, we represent a case series of four patients with HvRT treated with A + AVD (Table 1). The first patient was a 61 y/o male who was initially diagnosed with Rai stage 1 CLL at the age of 58 when he was found to have an enlarged left axillary lymph node biopsy. Cytogenetics was notable for trisomy with additional clonal abnormalities including inversion 3, deletion 6q, deletion 11q, and deletion 13q. Immunoglobulin heavy chain variable region (IGHV) mutational analysis was unmutated. The patient was initiated on ibrutinib and venetoclax due to progressive symptomatic lymphadenopathy for 12 months of fixed duration therapy. At the end of 12 months, he achieved undetectable minimal residual disease (uMRD) in both peripheral blood and bone marrow. Eight months after completion of treatment, the patient began to develop fever, night sweats, weight loss, and progressive cervical lymphadenopathy. A bone marrow biopsy was performed which did not demonstrate morphologic or immunophenotypic evidence for CLL. However, on core biopsy, there were focal areas of rare atypical larger cells that were CD30 positive concerning for the diagnosis of HvRT. A PET/CT was subsequently performed which demonstrated multiple hypermetabolic lymph nodes present above and below the diaphragm as well as heterogeneous increased splenic activity was concerning for splenic involvement, and multiple hypermetabolic bone marrow foci in the spine, ribs, osseous pelvis, and bilateral femurs were concerning for osseous involvement (Figure 1). A left axillary excisional lymph node biopsy confirmed HvRT. There was no evidence of concurrent CLL. The patient was subsequently initiated on A + AVD. After two cycles an interim PET/CT was consistent with a complete metabolic remission, Deauville score 2. He went on to complete 6 cycles of A + AVD; however, for cycle 6, brentuximab vedotin dose was reduced to 0.9 mg/kg for grade 3 neuropathy. An end of treatment PET/CT was once again consistent with CR, Deauville score 2. The patient remained in remission from both CLL and HvRT to date, now 40 months since completion of A + AVD.

Patient 2 was diagnosed with Rai stage II CLL at the age of 34 after he was found to have painless cervical lymphadenopathy. Cytogenetics was notable for trisomy 12, gain of chromosome 18, and gain of chromosome 19. IGHV was found to be unmutated. After 5 years of surveillance, the patient began to develop symptoms of progressive symptomatic lymphadenopathy with resultant left lower extremity lymphedema. An ultrasound demonstrated a large lymph node in the left groin measuring 5.3 × 3.9 × 3.2 cm. The patient was subsequently initiated on acalabrutinib 100 mg twice a day. Two months after starting acalabrutinib, the patient developed progressive bilateral inguinal lymphadenopathy. A PET/CT was performed which demonstrated multiple hypermetabolic mediastinal, abdominal, retroperitoneal, common iliac, internal iliac, external iliac, and left inguinal hypermetabolic lymphadenopathy. The left inguinal lymph node was most hypermetabolic, measuring 5.0 × 4.4 cm with an SUV max of 14.8. An excisional left inguinal lymph node biopsy demonstrated the presence of classic Hodgkin Reed–Sternberg cells scattered throughout the background of small lymphocytic lymphoma, consistent with HvRT. The patient was subsequently initiated on A + AVD. After two cycles, an interim PET/CT demonstrated markedly decreased uptake and size of lymphadenopathy, consistent with Deauville score 4. The patient was continued on A + AVD for an additional 4 cycles but however had to undergo dose reduction of BV to 0.9 mg/kg due to neuropathy starting with cycle 3 and then 0.7 mg/kg starting with cycle 5, and then, brentuximab was omitted on C6D15 due to ongoing grade 3 neuropathy. An end of treatment PET/CT was consistent with a partial response, with mild FDG activity noted in a periaortic lymph node (SUV max 2.2), left external iliac lymph node (SUV max 3.5), and left pelvic side wall lymph node (SUV max 3.5) (Figure 1). The patient subsequently underwent a left external iliac excisional lymph node biopsy which was consistent with residual CLL/SLL without any evidence of cHL present. Based on the results of the biopsy with residual CLL, the patient was restarted on acalabrutinib 100 mg bid, and remains on therapy today without any evidence of recurrent cHL, now 42 months since completion of A + AVD.

Patient 3 was diagnosed with Rai stage III CLL at the age of 60 after being found to have peripheral blood lymphocytosis and symptomatic left cervical lymphadenopathy. Cytogenetics was notable for deletion 17p in 10% of cells as sole cytogenetic abnormality, and the IGHV mutational status was found to be unmutated. The patient was initially treated with 7 cycles of FCR achieving a complete remission. Approximately 6 years after her initial diagnosis, the patient developed recurrent symptomatic lymphadenopathy and a bone marrow biopsy confirmed recurrent CLL. The patient was initiated on ibrutinib 420 mg daily for recurrent disease. One month after initiating ibrutinib, the patient developed recurrent low grade-fevers and was diagnosed with pulmonary aspergillosis and initiated on antifungal treatment. Despite treatment for aspergillosis, the patient continued to have low-grade fevers, and symptomatic progressive axillary lymphadenopathy. A PET/CT was performed which demonstrated multiple hypermetabolic lymph nodes present above and below the diaphragm. Focal uptake at the right aspect of the sacrum was also identified concerning osseus involvement. An excisional right axillary lymph node biopsy was performed which demonstrated involvement by cHL (60%) arising in association with SLL with plasmacytic differentiation (40%), most consistent with HvRT. The patient was subsequently initiated on A + AVD. After two cycles, an interval PET/CT demonstrated interval decrease in size and FDG uptake in the lymphadenopathy above and below the diaphragm, consistent with Deauville 3. An end of treatment PET/CT after 6 cycles of A + AVD was concerning for progression with interval increased size and activity of multistation lymphadenopathy (Figure 1). A right cervical lymph node biopsy was performed which demonstrated persistent cHL within the background of concurrent CLL. The patient went on to receive salvage treatment for cHL first with gemcitabine, dexamethasone, and cisplatin (GDP), then nivolumab, but unfortunately had disease progression on both treatments. She finally was administered bendamustine and BV. After completion of bendamustine and BV, a repeat CT scan demonstrated interval decrease in multi-station lymphadenopathy. A repeat bone marrow biopsy was consistent with persistent CLL, involving 80% of the bone marrow, but no evidence of cHL. The patient's course was complicated by persistent pancytopenia and recurrent febrile neutropenia, eventually leading to her death from complications of pancytopenia.

Patient 4 was a 45-year-old male who was admitted to the hospital after a one-month history of progressive cough and shortness of breath. A CT scan of his neck, chest, abdomen, and pelvis demonstrated bilateral conglomerate nodal masses in the right neck measuring 6.8 × 3.7 × 8.5 and the left measuring 4.3 × 4.7 × 8.7 cm, enlarged mediastinal and axillary lymph nodes, the largest was a prevascular lymph node measuring 2.8 cm, and a large splenic mass measuring up to 6.7 cm. An excisional left cervical lymph node biopsy was performed which demonstrated nodular sclerosis cHL. A staging bone marrow biopsy demonstrated a normocellular marrow with trilineage hematopoiesis, however there was 2.5% involvement by a CD5+, CD19+, CD20 (dim), CD23+ monoclonal b cell population, most consistent with CLL. Additional studies performed on the lymph node biopsy demonstrated the presence IgH and IgK rearrangements, which was thought to be most likely from concurrent CLL. The patient was initiated on A + AVD, an interim PET/CT after two cycles of treatment demonstrated interval decrease size and FDG avidity of hypermetabolic lymph nodes. The patient went on to complete 6 cycles of A + AVD, and an end of treatment PET/CT demonstrated interval development of hypermetabolic multistation lymphadenopathy, consistent with disease progression (Figure 1). An excisional left cervical lymph node biopsy demonstrated recurrent cHL, with concurrent CLL. He subsequently went on to receive 3 cycles of rituximab, ifosfamide, carboplatin, and etoposide (R-ICE) but unfortunately had disease progression. He subsequently completed pretransplant salvage radiation therapy to areas of disease involvement, followed by a matched unrelated donor allogeneic stem cell transplant with a myeloablative conditioning regimen consisting of fludarabine and busulfan. Unfortunately, his transplant course was complicated by acute graft versus host disease of the colon as well as pulmonary aspergillosis causing hypoxic respiratory failure, and he ultimately died from complications of respiratory failure.

## 3. Discussion

Here, we present the first reported case series of patients with HvRT treated with front-line A + AVD. Traditionally, for patients with HvRT, treatment has been adapted from the experience of patients with de-novo cHL, with the administration of ABVD chemotherapy. Initial retrospective reports from MD Anderson demonstrated a median overall survival (OS) of less than one year [15]. More recent reports have demonstrated slightly improved overall response rates (ORR) for patients treated with ABVD, ranging between 40 and 60%, with a median overall survival ranging between 2 and 4 years [16, 17]. This is significantly inferior to that of patients with advanced stage de-novo cHL, where ABVD yields a complete response rate of approximately 80%, with a median OS greater than 10 years [18–20]. A recent report demonstrated two-year PFS and overall survival OS from HvRT to be 47% and 57%, respectively. However, for patients treated with curative-intent ABVD/ABVD-like therapy, 2-year PFS and OS were 70% and 74%, respectively [21]. Moreover, patients with HvRT who have been previously treated with fludarabine have particularly poor outcomes, with median OS estimated to be less than one year [17]. Outcomes for patients treated with targeted therapy prior to HvRT are unknown; however, for patients with DLBCL-RT, there has not been an appreciable difference in incidence of RT or survival compared to traditional chemoimmunotherapy approaches [22].

Our patients had high-risk CLL, with high-risk CLL-IPI score, as well as high-risk feature including by cytogenetic abnormalities including trisomy 12, deletion 17p, and complex cytogenetics (Table 1). Furthermore, three patients had an unmutated IGHV. One patient had previous treatment with FCR and then subsequently BTKi; one of the patients was treated solely with BTKi, one with both BTKi and venetoclax; and one patient was treatment naive. At the time of diagnosis of HvRT, all patients were found to have an advanced stage disease. Three patients were found to have type 1 transformation, with concurrent CLL, while one patient had a type 2 transformation, without concurrent CLL. Two patients were positive for EBV by immunohistochemistry, while two were negative. All patients were treated with 6 cycles of A + AVD, without unexpected side effects related to treatment. Two of the patients required reduction of BV secondary to neuropathy, but this did not correlate with reduced efficacy. Two patients achieved a complete remission after completion of A + AVD, while two patients had a primary refractory disease, with disease progression at the end of treatment. The two patients who responded had lower IPS scores, which may have indicated a lower-risk disease. Interestingly, in our small series, interim PET/CT did not seem to correlate with end of treatment response. The two patients who achieved a CR remain in remission today without further HvRT directed therapy, while those who had disease progression ultimately died from complications of disease and/or subsequent treatment (Table 2).

The molecular origins of HvRT and CLL remains are still unclear. Xiao et al. demonstrated that clonally related cHL was more common for patients with Type II HvRT (53% vs. 29%); however, EBV status did not correlate with clonality. Furthermore, prognosis appeared independent of clonality, or type 1 or type 2 HvRT, with a median OS of only 44 months. The only factor that predicted survival was advanced age, >70 [10]. In our series, the response did not appear dependent on the type of HvRT; however, for the two patients who did response both were EBV-positive. BV has demonstrated activity in patients with EBV positive CD30+ lymphomas, where it can produce durable responses with reduction of EBV viral loads [23, 24]. Thus, it may be more effective in combination with chemotherapy for patients with EBV positive HvRT.

One of the significant challenges of treating with patients with HvRT is that patients may have continued active CLL that requires treatment, and traditional cHL directed chemotherapy is not optimal treatment for CLL In patients with DLBCL-RT, the combination of venetoclax plus dose-adjusted rituximab, etoposide, prednisone, vincristine, cyclophosphamide, and doxorubicin (VR-EPOCH) appears to lead to improved response rates for patients with RT, while also yielding high rates of undetectable one marrow minimal residual disease for CLL [25]. Thus, novel treatment strategies that incorporate CLL directed therapy in combination with chemotherapy, including brentuximab, may be more effective in treating HvRT. Furthermore, the use of checkpoint inhibitors has dramatically improved outcomes for patients with relapsed/refractory cHL [26, 27]. The combination of BV and nivolumab has been found to demonstrate high response rates and durable remissions for patients with R/R cHL, even in those who has refractory disease [28]. More recently, the SWOG 1826 trial demonstrated improved PFS of the combination of nivolumab + AVD, versus A + AVD for patients with treatment naïve advanced stage cHL [29]. Thus, incorporating the administration of checkpoint inhibitors in combination with BV-based chemotherapy may be an alternative treatment strategy that could improve outcomes.

## 4. Conclusion

HvRT is a rare complication for patients with CLL, with an overall poor prognosis, compared to de-novo cHL. We present the first case series of four patients with HvRT treated with A + AVD. In our series of 4 patients, two patients treated with A + AVD for HvRT had durable remissions of 40 and 42 months, while two patients had disease progression and ultimately died. Ongoing studies to determine improved treatment strategies for patients with HvRT are needed. Incorporating CLL directed therapy or checkpoint inhibitors in combination with BV-based chemotherapy may be potential alternative treatment strategies.

## Figures and Tables

**Table 1:** Patient characteristics.

	Patient 1	Patient 2	Patient 3	Patient 4
Age at transformation, years	61	43	67	44
Gender	Male	Male	Female	Male
Rai stage	II	II	III	III
CLL-IPI	5	3	10	N/A
Cytogenetics	Trisomy 12, inversion 3, deletion 6q, deletion 11q, deletion 13q	Trisomy 12; +18; +19	Deletion 17p; +8q; +11q; deletion 13q	Normal
IGHV mutational status	Unmutated	Unmutated	Unmutated	N/A
Time to transformation	41 months	80 months	78 months	0 Months
MRD status of CLL at time of transformation	uMRD	+MRD with concurrent CLL	+MRD with concurrent CLL	+MRD with concurrent CLL
Prior CLL therapy	Ibrutinib + venetoclax	(1) Ibrutinib	(1) FCR	None
		(2) Acalabrutinib	(2) Ibrutinib	
			(3) Acalabrutinib	
Type I or II	Type II	Type I	Type I	Type I
EBV status	Positive	Positive	Negative	Negative
RS cell immunophenotype	CD30+; CD15+; MUM1+; PAX5 (dim); CD20-, CD5-	CD30+; CD15+; MUM1+; PAX5 (dim); CD20-, CD5-	CD30+; CD15+; MUM1+; PAX5 (dim); CD20-, CD5-	CD30+; CD15+; MUM1+; PAX5 (dim); CD20-, CD5-
Ann arbor stage	IVB	IIIB	IVB	IIIB
LDH (U/L)	248	189	276	302
Bone marrow involvement	Yes	No	No	No
>1, extranodal site	No	No	No	No
IPS score	2	1	3	3

CLL-IPI = international prognostic index for chronic lymphocytic leukemia; IGHV = immunoglobulin heavy chain variable; MRD = minimal residual disease; uMRD = undetectable minimal residual disease; CLL = chronic lymphocytic leukemia; EBV = Epstein–Barr virus; RS = Reed–Stenberg; LDH = lactate dehydrogenase; IPS = international prognostic score; FCR = fludarabine, cyclophosphamide, and rituximab.

**Table 2:** Treatment summary.

	Patient 1	Patient 2	Patient 3	Patient 4
# of cycles of A + AVD	6	6	6	6
Dose reduction	Brentuximab dose reduced starting cycle 6 for neuropathy	Brentuximab dose reduced starting cycle 4 for neuropathy	No	No
Interim PET response	CR, Deauville 2	PR, Deauville 4	CR, Deauville 3	PR, Deauville 4
End of treatment response	CR	CR	PD, Deauville 5	PD, Deauville 5
Subsequent HvRT therapy	No	No	(1) GDP	(1) R-ICE
			(2) Nivolumab	(2) Allogeneic stem cell transplant
			(3) Bendamustine + brentuximab	
Residual CLL after completion of treatment	No	Yes	Yes	Yes
Additional CLL directed therapy	No	Acalabrutinib	(1) Rituximab	No
			(2) R-CVP	

A + AVD = brentuximab vedotin, doxorubicin, vinblastine, and dacarbazine; PET = positron emission Ttomography; HvRT = Hodgkin lymphoma variant of Richter's transformation; CLL = chronic lymphocytic leukemia; CR = complete response; PR = partial response; PD = disease progression; GDP = gemcitabine, dexamethasone, and cisplatin; R-CVP = rituximab, cyclophosphamide, vincristine, and prednisone; R-ICE = rituximab, ifosfamide, carboplatin, and etoposide.

## Data Availability

All data related to this work are in the manuscript.
